# Telomerase: The Devil Inside

**DOI:** 10.3390/genes7080043

**Published:** 2016-07-29

**Authors:** Mukesh Kumar, Andre Lechel, Çagatay Güneş

**Affiliations:** 1 Department of Urology, Ulm University, 89081 Ulm, Germany; mukesh.kumar@uniklinik-ulm.de; 2 Department of Internal Medicine I, Ulm University, 89081 Ulm, Germany; andre.lechel@uni-ulm.de

**Keywords:** telomerase, telomere, cancer, stem cells

## Abstract

High telomerase activity is detected in nearly all human cancers but most human cells are devoid of telomerase activity. There is well-documented evidence that reactivation of telomerase occurs during cellular transformation. In humans, tumors can rely in reactivation of telomerase or originate in a telomerase positive stem/progenitor cell, or rely in alternative lengthening of telomeres, a telomerase-independent telomere-length maintenance mechanism. In this review, we will focus on the telomerase positive tumors. In this context, the recent findings that telomerase reverse transcriptase (*TERT*) promoter mutations represent the most common non-coding mutations in human cancer have flared up the long-standing discussion whether cancer originates from telomerase positive stem cells or telomerase reactivation is a final step in cellular transformation. Here, we will discuss the pros and cons of both concepts in the context of telomere length-dependent and telomere length-independent functions of telomerase. Together, these observations may provoke a re-evaluation of telomere and telomerase based therapies, both in telomerase inhibition for cancer therapy and telomerase activation for tissue regeneration and anti-ageing strategies.

## 1. Telomerase Activity Is Required for Continuous Proliferation Capacity of Human Cells and Is Regulated through *TERT* Gene Expression

The activity of telomerase is necessary to overcome the end replication problem [1]. This enzyme extends the telomeric DNA at the very tips of linear chromosomes by the addition of a short GT-rich repetitive DNA sequence thus preventing loss of genetic material in proliferating cells [1,2,3]. Emerging evidence indicates additional roles of telomerase beyond this prime function (see below and [4] for a review on this aspect). In humans, telomerase activity is progressively downregulated during embryogenesis and only adult stem cell compartments still maintain low levels of telomerase activity [5,6,7,8]. Induction of telomerase was also observed in a subset of highly proliferating cells—such as proliferating B- and T-cells and the regenerating hepatocytes [9,10,11,12].

It is now well documented that telomerase activation is primarily regulated at the transcriptional level of its catalytic subunit, telomerase reverse transcriptase (TERT) [13,14,15]. In recent years, a number of additional factors, including dyskerin, TCAB1, and NOP10 have been identified to be constantly or transiently associated with the telomerase complex and have important functions in telomerase recruitment to telomeres or subcellular localization of the telomerase complex [16,17,18]. Although telomerase activity could be detected in the majority of human cancers [19], it is worth mentioning that about 10%–15% of human tumors were devoid of telomerase activity. Tumors that lack telomerase activity maintain their telomere length via a recombination-based mechanism (alternative lengthening of telomeres, ALT) [20]. Thus, telomerase or another mechanism for telomere maintenance is required for continuous tumor cell proliferation. Importantly, the prominent occurrence of telomerase in human cancers motivated the development of telomerase inhibitors to suppress tumor growth [21,22,23,24,25]. As a flaw, however, anti-telomerase therapy may provoke ALT mechanism in cancer cells [26].

## 2. Regulatory Mechanisms Involved in *TERT* Gene Regulation in Normal and Tumor Cells

In the light of these findings and considering the close correlation of *TERT* gene expression and telomerase activity with cancer, investigations focused on deciphering the underlying mechanisms that govern *TERT* promoter activity. A number of factors have been identified to be involved in the regulation of *TERT* expression. These factors include c-Myc and its antagonist Mad1, the receptors for the hormones estrogen and progesterone, AP-1 (Activator Protein 1), NF-κB (nuclear factor ‘kappa-light-chain-enhancer’ of activated B-cells), Rb/E2F factors (Retinoblastoma tumor suppressor protein/ E2 factor family of transcription factors), CEBP-α (CCAAT/Enhancer Binding Protein Alpha) and CEBP-β (CCAAT/Enhancer Binding Protein Beta) (see [27,28] for a comprehensive review on *TERT* promoter regulation). In the stem cell context, Wnt/β-catenin pathway and Kruppel-like factor 4 (KLF4) were described to regulate *TERT* gene expression and telomerase activity [29,30].

**Figure 1:**
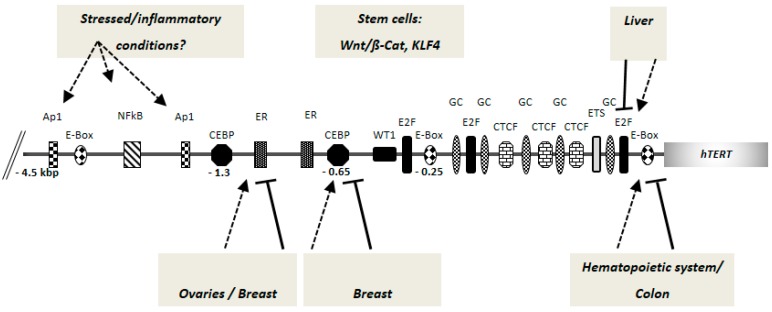
Putative and bona fide *cis*-regulatory elements of human telomerase reverse transcriptase (*hTERT*) gene promoter. Current research indicates that different factors can act in a tissue specific manner for *hTERT*/telomerase regulation. A 4.5 kbp *hTERT* promoter fragment upstream of the AUG start codon is shown (not drawn to scale). The numbers denote the distance from the AUG start site. The arrows indicate activation and the lines indicate repression of *hTERT* promoter by the same binding site, depending on the occupation by the respective tissue-specific regulatory factors (see text for details). The mode of action by the stem cell specific factors is not fully clear. E-box: c-Myc/Mad-family factor binding sites; E2F: E2 factor family transcription factor binding sites; GC: GC-box binding sites; ETS: ETS-domain binding sites; CTCF: CTCF factor binding sites; CEBP: CEBP family factor binding sites; ER: estrogen receptor binding sites; Ap1: Ap1 factor binding sites; NFκB: NFκB factor binding site; WT1: binding site for the Wilms’ tumor 1 transcription factor.

Although these factors were shown to regulate human telomerase reverse transcriptase (*hTERT*) promoter and telomerase activity in experimental systems, it remains to be shown whether these factors and mechanisms are also responsible for high telomerase activity in human cancer. Based on the current knowledge, it seems tempting to speculate that the same *cis*-regulatory elements can act as a site of positive and negative regulation of *hTERT* gene expression depending on the proliferation status, at least in some tissues; e.g., E-box binding by c-Myc activates the *hTERT* promoter whereas the binding of its counterpart Mad1 to the E-box sequences results in *hTERT*/telomerase downregulation [15,31,32]. Similarly, CEBP-α represses *hTERT* in normal breast cells, but CEBP-β activates it in breast cancer cells by binding to the same regulatory sites [33]; in resting hepatocytes, Rb factors repress *hTERT* expression whereas E2F2/E2F7 factors are involved in *hTERT* promoter activation in proliferating hepatocytes by binding to the same sequence [12]. Interestingly, ETS2 transcription factor was described both as a positive and negative regulator of wild-type *hTERT* promoter by regulating promoter activity through a native ETS binding site [32,34,35,36]. Recent data indicates that the ETS factor GA-binding protein alpha chain (GABPA) binds to the mutated *hTERT* promoter (generating a new potential ETS binding site), likely in combination with the juxtaposed native ETS binding site (Figure 1) leading to elevated *hTERT* expression and telomerase activation in cancer cells [37].

Beyond this assumption, several additional mechanisms were described for *hTERT* activation in human tumor cells:

- **Alteration of regulatory factors:** In line with the above regulatory factors, changes in the expression levels of the respective factors could account for the upregulation of *hTERT* gene in cancer cells. One example may be the increase of c-Myc due to gene amplification in breast cancer [38]. Another potential mechanism could be the loss of negative regulatory factors resulting in derepression of *hTERT* transcription, e.g., loss of the candidate tumor suppressor Mad-1 expression in many cancer types or loss of the Wilms’ tumor suppressor 1 (WT1) in clear cell renal cell carcinoma [15,39].
- **Epigenetic regulation of promoter activity:** Another form of *hTERT* upregulation is through demethylation of histones proximal to the promoter region, imitating the low density of trimethylated histones seen in embryonic stem cells [40]. This permits the recruitment of histone acetyltransferases (HATs) allowing the transcription of the gene [41]. However, current data indicate a complex epigenetic regulation of *hTERT* promoter activity: it has been demonstrated that methylation status is crucial for the regulation of *hTERT* gene expression [42,43] and that the chromatin remodeling factor CTCF (CCCTC-binding factor) can bind to the *hTERT* promoter depending on the methylation status and suppress its expression [44]. On the other hand, demethylation of specific regions on *hTERT* promoter caused downregulation of *hTERT* gene expression, telomerase activity, and telomere shortening, indicating that a certain level of methylation is required for *hTERT* promoter activation [45]. In the same line, aberrant *hTERT* promoter hypermethylation correlates with elevated *hTERT* gene expression in the majority of non-infant Sonic-Hedgehog subgroup medulloblastoma tumors [46] whereas *hTERT* promoter methylation was shown to correlate with reduced *hTERT* gene expression in B-cell lymphocytic leukemia and childhood acute lymphoblastic leukemia (ALL) [47,48]. Alterations in the epigenetic pattern of *hTERT* promoter, e.g., by point mutations, may also lead to a deregulation of promoter activity [49,50]. In this line, recurrent genomic rearrangements in the chromosomal region at 5p15.33 proximal of the *hTERT* gene induced massive chromatin remodeling and DNA methylation leading to the transcriptional upregulation of *hTERT* expression and telomerase activity in high-risk neuroblastomas [51]. Future studies will clarify the exact mechanisms and the potential tissue-specific factors involved in the methylation-dependent *hTERT* promoter regulation.
- **Amplification and genomic rearrangements**: Structural and numerical changes in the genome organization can result in *TERT* promoter activation. Amplification of the *hTERT* gene locus was proposed as a mechanism for increased *hTERT* mRNA levels and telomerase activity [52,53,54]. It should be noted however, that the elevated *hTERT* mRNA levels may not necessarily be attributable to increased *hTERT* transcription.
- **Promoter mutations:** Over the last few years, mutations in the *hTERT* gene promoter, first described in melanoma [55,56], were found in several tumor types, including bladder cancer (29%–90%), hepatocellular carcinoma (HCC) (30%–63%), melanoma (29%–73%), thyroid cancer (10%), and tumors from the central nervous system (43%–51%), representing one of the most frequent non-coding mutations in human cancer (see [57,58,59,60,61,62] for a comprehensive overview on *hTERT* promoter mutations). On the other hand, other tumor entities have a low frequency or no *hTERT* promoter mutations which are described up to the recent date, including testicular germ cell tumors, colorectal adenocarcinoma, pancreatic cancer, papillary thyroid cancers, and most types of leukemia [63,64,65] despite high telomerase activity in these tumors [66,67,68]. These observations indicate that other mechanisms, e.g., loss of negative regulatory factors or chromatin remodeling may be responsible for telomerase reactivation in the tumor types (e.g., loss of suppression: see above). Alternatively, tumors may originate from telomerase positive stem or progenitor cells (see below).

## 3. Telomerase Reactivation versus Telomerase Positive Stem Cell Hypothesis

**Figure 2:**
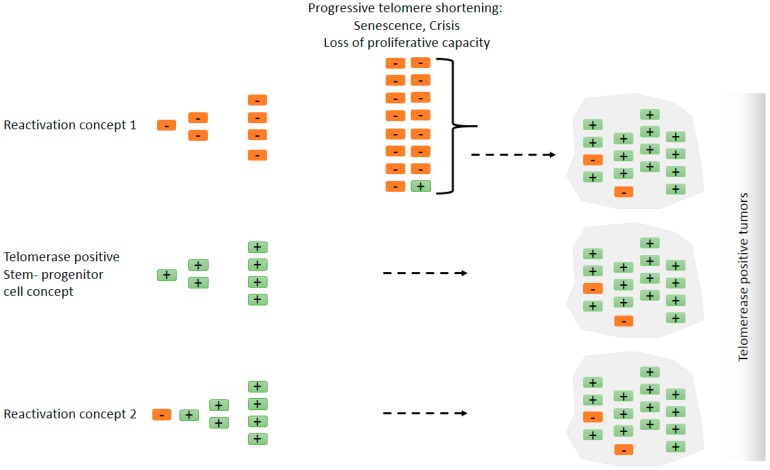
**Does telomerase reactivation occur in tumors, or do tumors originate from telomerase positive cells such as stem cells?** Simplified models explaining ‘detectable’ telomerase activity in cancer. To what extent the final tumor mass contains telomerase negative cells is unclear. There is evidence, however, that there is a subset of cells that are telomerase-positive cancer stem cells [92]. Orange rectangles with a negative mark represent telomerase negative cells and green rectangles with a positive mark represent telomerase positive cells. Reactivation concept 1: Telomerase negative cells with a tumorigenic mutation can proliferate but eventually enter senescence and crisis due to progressive telomere shortening. Cells that evade *hTERT* repression can continue to proliferate and constitute the major tumor mass. In this scenario, telomerase reactivation occurs late during tumorigenesis or it is not detectable because only a minority of patient-derived early lesion cells are telomerase positive. Stem/progenitor cell concept: The tumorigenic mutation, e.g., aneuploidy-inducing mutation [75], occurs in a telomerase positive stem/progenitor cell. In the presence of telomerase, cells continue to proliferate where telomerase activity may suppress telomere shortening during extended proliferation and cellular differentiation [76] and/or confer telomere-length-independent survival advantage. Reactivation concept 2: Telomerase reactivation occurs early in tumorigenesis, e.g., by *hTERT* promoter mutations or by loss of negative regulatory mechanisms, and telomerase activity is high at all stages. Telomerase reactivation may precede other tumorigenic events. It is important to note that the alternative models proposed here are non-exclusive. The three mechanisms are very likely to take place under different scenarios and different cancer types.

It is a long-standing debate whether telomerase is reactivated during tumor progression or if tumors initiate from telomerase-positive stem cells [69,70,71,72,73,74,75,76,77,78]. The telomerase reactivation concept is based on two early observations: (i) Telomerase activity is detectable in human tumors and transformed cells but not in most primary human cells, e.g., normal human fibroblasts or embryonic kidney cells [19,79,80]; (ii) in the absence of telomerase, telomere shortening coincides with the induction of the ‘cellular senescence’ phenotype in cells with functional checkpoints [81,82]. Consequently, cells devoid of functional p53 and Rb proteins overcome the senescence barrier but telomeres continue to shorten until a ‘crisis’ checkpoint. Cells that survive the crisis checkpoint possess telomerase activity [80]. In this concept, telomere shortening in the absence of telomerase acts as a tumor suppressor mechanism but dysfunctional telomeres in the absence of functional checkpoints can initiate cancer formation due to increased genome instability leading to oncogene activation/loss of tumor protective factors [83]. Studies in a variety of human and mouse cancer models, including breast cancer, HCC, and prostate cancer provided evidence for the telomere hypothesis [77,84,85,86,87,88,89]. In this model, the activation of telomerase is an essential instrument to stabilize telomere length and may function as an active driver of tumor progression [77]. This telomere hypothesis implies that the reactivation of telomerase is a late step during cellular transformation supported by low/undetectable telomerase activity in normal tissues and premalignant lesions [19] (Figure 2, reactivation concept 1).

This idea was questioned, arguing that telomerase activity may be present in a few cells in premalignant lesions, difficult to detect by the conventional methods, e.g., telomere repeat amplification protocol (TRAP). Instead, tumors may arise from telomerase positive stem cells [71,74]. There is emerging evidence that stem cells accumulate mutations during ageing and can be the cancer-initiating cell type [90,91]. Since stem and progenitor cells possess telomerase activity, these cells do not require a re-activation of telomerase during tumorigenesis. There is evidence from a number of tumor types (such as most leukemia or testicular tumors) supporting this hypothesis (Figure 2, stem/progenitor cell concept). In fact, tumors can originate from different sources within the same tissue, depending on the genetic event and on the activated pathways initiating tumorigenesis [91].

The new findings that promoter mutations can be found at early steps during carcinogenesis (Reactivation concept 2) open up new perspectives on this debate. The most noticeable conclusion from these findings is that, irrespective of the reactivation mechanism, the data support the idea that at least a subset of tumors originate from an initially telomerase negative cell. Remarkably, *hTERT* promoter mutations are frequently detected in cancers with low self-renewal rates [55,56,57,58,59,60,61,62] whereas they are extremely rare in tumors originating from highly proliferating tissues [63,64,65]. We suggest that *hTERT* promoter mutations may account for both reactivation concepts 1 and 2. For example, late telomerase reactivation can result from *hTERT* promoter mutations in thyroid cancer and gliomas (reactivation concept 1) but *hTERT* promoter mutations in bladder and some skin tumors can result in reactivation of telomerase at an early stage of tumorigenesis (reactivation concept 2). However, it should be noted that mutations in the *hTERT* promoter region are not found in all telomerase positive bladder cancer or HCC samples and are missing in some tumor types altogether (e.g., pancreatic cancer) despite high levels of telomerase activity in these tumors indicating that other telomerase activating mechanisms must account for increased *hTERT* expression and telomerase activity.

One interesting aspect remains as to whether reactivation of telomerase is an early or late event during tumorigenesis (Figure 2, reactivation concept 1 versus reactivation concept 2). As discussed above, there is well-documented evidence from human cancers that telomerase reactivation is rather a late event during tumor progression (telomere hypothesis) supported by cell culture experiments [80] although difficult to prove formally in progressing tumors. The new findings show that *hTERT* promoter mutations occur already at early stages and across all grades of bladder cancer [93,94,95,96]. Similarly, *hTERT* promoter mutations were frequent in HCC, detectable early in the process of tumor development and were already present in dysplastic macronodules but not in cirrhotic liver [58], indicating that reactivation of telomerase represents a very early event in the multistep progress of carcinogenesis and might be a key driver. Telomerase reactivation occurring early during tumorigenesis would generate a ‘stem-like’ phenotype preventing telomere shortening and the senescence pathway. Interestingly, human embryonic stem cells that were genetically modified to carry the common *TERT* promoter mutations failed to suppress telomerase activity during differentiation and maintained telomere length, providing evidence for this idea [76].

Taken together, the activity/reactivation of telomerase in cancer supports its classical role on telomere length maintenance. Telomerase has also been implicated in non-canonical functions which may contribute to cancer cell survival in a telomere length-independent fashion.

## 4. Providing Cellular Survival Advantage Telomere Length-Independent Mechanisms

There is well-documented evidence for telomere length-independent roles of telomerase, both under physiological conditions and in cancer cells. These non-canonical functions include providing growth advantage by activating cellular growth factor pathways, improved ribosomal biogenesis or improved stem cell functionality by modulating gene expression [97,98,99,100,101,102,103,104,105], suppressing apoptosis and/or telomere replication stress [106,107,108] and promoting cell adhesion and migration [109]. It was shown that a catalytically inactive *hTERT* can promote tumor formation, supporting a telomere length-independent functional role of telomerase in tumorigenesis [110]. Contrary to these findings, by analyzing mouse TERT (mTERT) or mouse telomerase RNA (mTR) knockout mice, Greider and colleagues did not find evidence for telomere length-independent roles of telomerase: (i) Telomerase knockout mTERT-G1 and mTR-G1 mice with long telomeres did not show significant differences in transcriptional profiling or in DNA damage responses compared to wild-type mice [111]; and (ii) phenotypes in *mTERT*+/− and *mTERT*−/− mice were similar to the phenotypes observed in mTR+/− and mTR−/− mice and were linked to progressive telomere shortening [112]. Greider and colleagues also questioned the previous observations that indicated a transcriptional regulation of the Wnt/β-catenin pathway by the telomerase protein component TERT [103,112]. They argued that the ectopic TERT that was used in some of the above mentioned studies may act non-specifically [112]. It remains to be clarified in future studies whether there are species-specific differences in the function of telomerase components. Interestingly, an increasing number of *hTERT* splice variants have been identified in human cells, some of them lacking the catalytic activity region. Alternative splicing could induce changes in expression intensity, but splice variants can also act as dominant negative proteins to counteract wild-type telomerase function, both canonical and non-canonical [107]. One prominent isoform is the β-deletion variant lacking the exons 7 and 8, creating an activity-deficient protein that was detected both in normal and cancer cells [113]. It is therefore conceivable that the canonical and non-canonical functions are exerted by different splice variants. This idea opens up the opportunity that modulating the levels of the splice variants could be a tool to target telomerase activity in cancer cells specifically.

## 5. Final Conclusions

High-throughput sequencing technologies have provided a new insight into the mechanisms of telomerase activation in cancer cells. Whole genome sequencing analyses revealed highly prevalent *hTERT* promoter mutations in human cancer. The two key hot-spot mutations (positions C228T and C250T in *hTERT* promoter region, corresponding to positions −124 and −146 upstream of the ATG transcription start site, respectively) represent the most common non-coding mutations found in human tumors [59,114,115,116]. These mutations result in the activation of *hTERT* gene expression, much in line with the well-documented evidence that reactivation of telomerase occurs during cellular transformation. The reactivation concept is closely linked to the classical view that the downregulation of telomerase is a cancer protective mechanism by leading to telomere shortening and, eventually, to cellular senescence in checkpoint proficient cells, whereas the activation of telomerase is required to stabilize telomere length after telomere dysfunction-driven genetic instability initiates tumorigenesis [80]. The findings support the concept that some tumors can arise from telomerase positive cells—such as stem cells—in humans. *TERT* promoter mutations at early steps during tumorigenesis (e.g., bladder cancer) may therefore generate a ‘stem-like’ cell that can accumulate further mutations without activating senescence checkpoints.

Emerging evidence over the last decade supports the idea that telomere length-independent functions of telomerase are also important for its function, both in normal and tumor cells. Interestingly, current research also revealed that telomeres may sense cellular stress (such as genotoxic stress, oncogenic or aneuploidy-inducing mutations) that result from harmful mutations that lead to genome instability and induce senescence in cells with intact checkpoints [108]. Although the mechanistic details of the ‘sensing’ process are yet to be revealed, this new function of telomeres, thought to be a result of accumulating replication stress at the telomeres, seems to be independent of telomere length. In this context, telomerase relieves this cellular protective mechanism by mitigating telomere replication stress and this function of telomerase apparently is separate from its telomere elongation activity [75,78,117]. In light of the recent discoveries hinting at novel, telomere length-independent roles of telomeres and telomerase, attempts at modulating telomerase activity to improve organ function and longevity must be seriously reconsidered. In this line, interfering with telomerase activity and its extracurricular functions for cancer therapy seems to be an attractive strategy again but new concepts need to be taken into account.
